# Clinical implications of intraoperative eABRs to the Evo®-CI electrode array recipients

**DOI:** 10.1016/j.bjorl.2021.04.012

**Published:** 2021-05-14

**Authors:** Fabiana Danieli, Ana Cláudia Mirândola Barbosa Reis, Eduardo Tanaka Massuda, Maria Stella Arantes do Amaral, Michel Hoen, Dan Gnansia, Miguel Ângelo Hyppolito

**Affiliations:** a Universidade de São Paulo, Faculdade de Medicina de Ribeirão Preto, Programa de Pós-Graduação do Departamento de Oftalmologia, Otorrinolaringologia, Cirurgia de Cabeça e Pescoço, Ribeirão Preto, SP, Brazil; b Oticon Medical, Departamento Clínico, São Paulo, SP, Brazil; c Universidade de São Paulo, Faculdade de Medicina de Ribeirão Preto, Departamento de Ciências da Saúde, RCS, Ribeirão Preto, SP, Brazil; d Universidade de São Paulo, Faculdade de Medicina de Ribeirão Preto, Departamento de Oftalmologia, Otorrinolaringologia, Cirurgia de Cabeça e Pescoço, Ribeirão Preto, SP, Brazil; e Oticon Medical, Department of Scientific and Clinical Research, Vallauris, France

**Keywords:** Cochlear implants, Auditory evoked potentials, Speech perception, Cochlear nerve, Electrically evoked auditory potentials

## Abstract

**Introduction:**

Electrically evoked auditory brainstem responses provide reliable clinical information to assist professionals in the auditory rehabilitation of cochlear implant users.

**Objective:**

This study aimed to investigate intraoperative evoked auditory brainstem response recordings in Evo®-cochlear implant electrode array recipients and its correlation with their behavioral levels and auditory performance.

**Methods:**

: This is a retrospectivey study. Intraoperative evoked auditory brainstem responses were recorded in adult Evo®-cochlear implant electrode array recipients. Wave V latencies, amplitudes and interpeak III–V intervals were recorded in three different electrode locations and compared to the sentence recognition scores obtained from subjects after six months of device use. Evoked auditory brainstem responses thresholds were also recorded and compared to the behaviorally determined levels of the subjects in the sound processor activation.

**Results:**

Evoked auditory brainstem responses thresholds were significantly correlated with both, behavioral T- and C-levels and they were recorded at audible electrical stimulation levels in all subjects. There was a significant correlation between interpeak III–V interval recorded in the apical electrode and sentence recognition scores of the subjects.

**Conclusions:**

Intraoperative evoked auditory brainstem responses can be used to establish audible levels for fitting the sound processor in Evo®-cochlear implant recipients and it could help professionals to plan further actions aiming to improve their auditory performance.

## Highlights

• Intraoperative electrically evoked auditory brainstem response thresholds were recorded at audible levels in Evo® recipients.
• Absent intraoperative electrically evoked auditory brainstem response is related to inferior cochlear implant outcomes in the short term.
• Longer interpeak III–V interval could be related to inferior cochlear implant outcomes.
• Evoked auditory brainstem response can drive professionals to plan further actions aiming to improve cochlear implantation outcomes.

## Introduction

Cochlear implantation has become the most viable alternative for the treatment of people with severe to profound sensorineural hearing loss who do not benefit from conventional hearing aids. In these cases, cochlear implants (CIs) can effectively stimulate the auditory system through the electric pulses, promoting auditory perception and enabling the development of other auditory perceptual abilities to the user.

Several technological advances have taken place in the cochlear implant field in the last decade, and, although many studies have shown improved speech perception outcomes in patients using current CI systems, there is considerable variability in auditory performance with the device use.^1^, ^2^ Additionally, features such as electrode placement, neural survival and neural health of implanted patients influence the amount of electrical charge required to elicit an auditory perception among them.^3^ Hence, determining the optimal psychophysical levels of electrical stimulation during the sound processor fitting, based on behavioral measures, mainly for young children or patients with special needs, is still a challenge for the audiologists.

Thus, the use of objective measures that could provide reliable clinical information about these issues in CI recipients have been increasingly studied in the literature. Many studies have been performed to identify objective measures that could determine electrical stimulation levels for the initial map of CI recipients,^3^, ^4^ even during CI surgery.^5^ Objective measurements more commonly used during CI surgery, such as electrically evoked compound action potential (eCAP), have thus far not provided statistically significant correlations for use in predicting T- and C-level programming for all patients.^6^, ^7^ Still, regarding auditory performance with CI, more central objective measures, such as cortical auditory evoked potentials, have been strongly correlated to it.^8^ However, this measure depends on the patient’s collaboration, becoming difficult for children or patients with special needs. In addition, this measure needs to be recorded in an awake patient, and it cannot be performed intraoperatively.

Electrically evoked auditory brainstem response (eABR) is of interest in this regard because it is an objective measure of the functionality and responsiveness of the neural pathway from the cochlea to the cochlear nucleus, as well as more central structures. It allows the evaluation of critical synchronous components of neural encoding,^9^ and, according to these authors, nerve survival and synchronous neural activity may contribute to CI performance. eABRs can also be recorded intraoperatively^10^ and easily adopted in the clinical routine.

Previous studies showed significant correlations between morphology and the presence of wave V, and speech perception outcomes.^9^, ^11^, ^12^, ^13^, ^14^, ^15^ Other studies compared eABR waves latencies and amplitudes and auditory outcomes in CI users, but with controversial results. Brown et al.^11^ found weak or absent correlations between speech perception and eABR thresholds or wave V latencies and amplitudes. These results were later supported by Firszt et al.^9^ and Lundin et al.^15^ In contrast, Gallégo et al.^16^, ^17^ studied 17 adults implanted with the Digisonic DX10 CI system and found that wave V absolute latencies and interpeak II–V and III–V intervals were positively correlated with phoneme recognition scores; stepwise multiple regression analysis involving all eABR variables indicated that the interpeak III–V interval was a powerful predictor of speech recognition scores. Although Gallégo et al.^16^, ^17^ found a strong correlation between interpeak III–V intervals and speech perception outcomes in Digisonic DX10 users, they only assessed postoperative eABR recordings. It is important to determine whether these results can be replicated intraoperatively in Evo® cochlear implant electrode array recipients, once it could help professionals to plan further actions aiming to improve the auditory performance of these patients.

The correlation between postoperative eABR recordings and behaviorally determined levels obtained from CI users has also been reported in the literature. Some studies showed that postoperative eABR thresholds and behavioral thresholds (T-levels) were significantly correlated,^18^, ^19^, ^20^ including in Digisonic SP recipients.^3^ A few studies compared eABR thresholds recorded in the operating room and behavioral levels in subjects implanted with other CI systems and found that intraoperative eABR thresholds were consistently higher than postoperative behavioral thresholds and near the comfort levels.^21^, ^22^ To the best of our knowledge, there have been no studies correlating eABR thresholds recorded in the operating room and behaviorally measured perceptual thresholds in Evo® cochlear implant electrode array recipients. In this cochlear implant system, unlike other implant technologies, a multimode grounding stimulation mode associated with a duration modulated pseudo-monophasic electrical pulses with a capacitive discharge compose the electrical stimulation pattern. Undurraga et al.^23^ reported that pulse shape and signal processing strategies have a significant impact on objective measures. Studying the correlation between intraoperative eABR thresholds and behaviorally determined levels may provide valuable information for the first fitting of the sound processor in these patients.

Thus, this study aimed to investigate intraoperative eABR recordings in Evo® cochlear implant electrode array recipients among different electrode location as well as its correlation with behavioral levels and auditory performance in subjects implanted with this device.

## Methods

The present study was conducted in a Brazilian Cochlear Implant Center, from 2018 to 2020. Subjects with post-lingual onset of severe to profound sensorineural hearing loss, who showed limited benefit with the use of hearing aids (sentence recognition scores lower than 50% in the best ear with amplification) and who underwent cochlear implant surgery with the Evo® cochlear implant electrode array were included in the study. Children ages up to 12-years old and subjects with cognitive or central disorders and auditory neuropathy were excluded. The study was approved by the Ethics Committee (number 422/2017).

Twenty-one subjects (9 females and 12 males) aged between 14 and 81 years old (mean = 47.74 ± 17.98 years) were included in the study. Table 1 shows their demographic characteristics. Hearing deprivation comprised the time spent without a hearing aid before CI implantation and duration of deafness comprised the time between the first diagnosis of hearing loss and CI surgery. Duration of CI use comprised the time between the CI activation and the day that SRI scores were obtained.

**Table 1:** Demographic characteristics of subjects.

Subject	Sex	Implanted ear	Age at cochlear implantation (yr)	Hearing deprivation (yr)	Duration of deafness (yr)	Duration of CI use (yr)	Etiology
1	M	R	65.3	18.2	28.1	0.6	Genetics
2	M	R	75.2	2.3	6.5	0.6	Unknown
3	F	R	14.0	4.1	5.6	0.8	Unknown
4	F	R	57.8	17.8	22.6	0.7	Unknown
5	F	R	57.5	2.1	11.0	0.6	Chronic Otitis
6	M	L	39.5	1.1	3.2	0.6	Cholesteatoma
7	F	L	60.1	4.7	5.0	0.8	Unknown
8	M	R	55.7	20.0	26.1	0.8	Unknown
9	M	R	46.0	0.1	30.0	0.6	Genetics
10	F	L	53.5	10.3	28.1	0.6	Unknown
11	F	L	23.7	0.1	14.9	0.7	Unknown
12	M	R	49.0	0.9	5.0	0.6	Chronic Otitis
13	M	R	67.8	2.8	40.2	0.6	Cholesteatoma
14	F	R	35.0	1.0	2.1	0.6	Unknown
15	M	R	26.7	0.5	1.1	0.7	Meningitis
16	F	R	58.7	1.6	2.0	0.6	Unknown
17	M	R	32.2	1.3	20.8	0.8	Unknown
18	F	L	45.4	3.1	3.3	0.6	Unknown
19	M	R	31.8	1.0	1.9	0.8	Meningitis
20	M	R	80.9	0.9	10.0	0.6	Ototoxicity
21	M	L	26.7	0.5	1.1	0.6	Meningitis

Sex: female (F), male (M); implanted ear: right (R), left (L); yr, year.

### eABR recordings

Intraoperative electrically evoked brainstem responses were recorded in all subjects undergoing CI surgery with the Digisonic® Evo device (Oticon Medical, Denmark). The recordings were performed in the operating room at the time of the CI surgery, with subjects reclined under a stretcher, under general anesthesia, immediately after the insertion of the electrode array inside the cochlea. The electrical stimuli were produced by the implant, using the Digistim USB interface and Digistim software system, version 1.9.15 (Oticon Medical, Denmark). The stimulus used for the eABR measures was a 70µs/70 CU (1.56 mA) pseudo-monophasic pulses with a capacitive discharge presented at a rate of 21 Hz. eABRs were recorded using the Eclipse EP25 equipment (Interacoustics A/S, Middelfart, Denmark) and Otoaccess software system, version 7.0 (Interacoustics A/S, Middelfart, Denmark). The recording system averaged 1200 sweeps and was filtered by a low-pass filter at 1500 Hz and a high-pass filter at 33 Hz/6 oct. An artifact rejection level of 40 µV and recording time window of 12 ms were used. The stimuli were synchronized with the recording system via a trigger line to allow synchronous averaging. Contact electrodes were placed on the vertex (positive), contralateral mastoid (reference), and ipsilateral cheek (ground). The impedance values of the recording electrodes were between 0 and 3 Ohms.

Three different intracochlear electrodes were tested: apical, mid, and basal electrodes (representing 500 Hz, 1000 Hz and 2000 Hz on the subject’s MAP). These frequencies are allocated, respectively, to the electrodes 18, 14 and 9 in the Evo® electrode array. The Evo® electrode array is composed of 20 full-band active electrodes, 24 mm of insertion length and it is associated with low levels of intracochlear traumas.^24^

**Figure 1:**
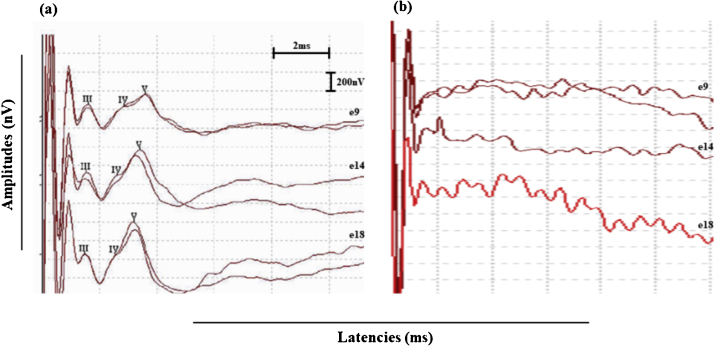
(a) Typical intraoperative eABR recordings classified as positive eABRs. Wave V could be recorded in two replications of the same stimulus condition in the three tested electrodes (electrodes 9, 14 and 18). (b) Negative eABRs recorded in the subject #21. Wave V could not be identified in both stimuli replications in one or more tested electrodes.

Wave identification was defined according to the morphological criteria proposed by Picton and Hillyard,^25^ as a series of positive peaks would occur between approximately 1–4 milliseconds (ms) and the latency of wave V would occur at approximately 3.5–4.0 ms. Wave I is not registered because it is embedded in stimulus artifact.^26^ The eABR recording was classified as “positive eABRs” when the wave V could be registered in two replications of the same stimulus condition in the three tested electrodes (Fig. 1a) and as “negative eABRs” when the wave V could not be identified in both stimuli replications in one or more tested electrodes (absent eABRs) (Fig. 1b). The peak to trough amplitude, measured in µV of each recorded eABR peak, was analyzed.

For the subjects who showed “positive eABR” recordings, wave V amplitudes, latencies and interpeak III–V intervals were recorded and compared across the different electrode locations and to the sentence recognition scores obtained from subjects after 6-months of CI use.

**Figure 2:**
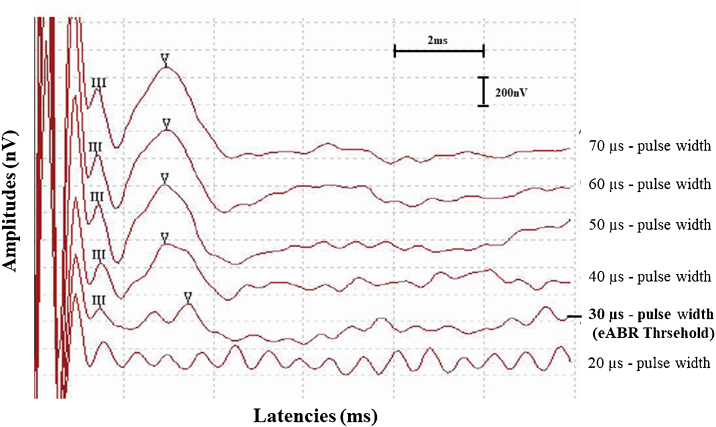
Example of eABR detection threshold recorded from subject 6 during the cochlear implant surgery. eABR threshold was defined as the lowest pulse width at which wave V could be detected (30 µs).

In addition, eABR thresholds were also recorded for these subjects and compared to their behaviorally determined C- and T-levels obtained in the sound processor activation. To achieve the eABR detection thresholds of the subjects, pulse width was decreased in 10 µs steps to a point where wave V could no longer be identified. Then, eABR threshold was defined as the lowest pulse width where wave V could be identified (Fig. 2).

### Behaviorally determined thresholds (T- and C-levels)

All subjects were fitted with a Saphyr Neo Collection speech processor one month after the CI surgery. A fixed stimulation rate of 500 Hz and a fixed amplitude of 70 computer units (CU = 1.56 mA) were adopted in the subject’s standard map. The multimode grounding stimulation mode^27^ and the duration-modulated pseudo-monophasic electrical pulses with passive discharge composed the electrical stimulation pattern used by the CI. In this mode of stimulation, electrical current simultaneously flows from one electrode within the cochlea to all remaining intracochlear electrodes and to one extracochlear electrode and this pattern of stimulation was also adopted for the eABR recordings. The only difference between the Digistim stimuli used to record the eABRs and in the sound processor was the stimulation rate. The first one stimulates at 21 Hz, the second one stimulates at 500 Hz.

Eight spectral maxims were selected in the Crystalis XDP speech coding strategy for all participants. This strategy corresponds to a multi-band spectral extraction associated with a multi-band compression function (XDP). A pre-selected number of electrodes were stimulated per an acquisition frame so that tonotopic electrical stimulation of the electrodes related to the higher energy of the input sound is performed.

Behavioral thresholds and maximum comfort levels were obtained in all subjects using the loudness scaling method by an audiologist trained on the sound processor programming software (Digimap SP, version 4.0.7, Oticon Medical, Denmark). To determine the T-level, the pulse width was increased in 2 µs steps and the subjects were instructed to indicate in the loudness scale when they started to perceive an auditory sensation. To obtain the C-level, the subjects were instructed to indicate in the loudness scale when the sound was comfortable. This procedure was performed to obtain repeatable T- and C-levels for each active electrode inserted in the cochlea and used to create the MAP for the subjects.

### Speech perception scores

A sentence recognition test^28^ was used to assess the subject’s open-set speech recognition in silence after 6-months of CI use. The test was composed of 60 sentences that were distributed into six lists of ten phonetically balanced sentences with up to seven words. Words with lexical and semantic meaning, such as nouns, adjectives, verbs, adverbs, and numerals were considered keywords and were scored with value 2. Words with grammatical meaning, such as articles, prepositions, conjunctions, pronouns, and interjections were scored using the value 1. As the lists of sentences were composed of different numbers of words, ranging from four to seven words and three to four keywords, the total score was multiplied by a correction value, expressed in percentage. This value varied according to the list, ranging from 1.11% to 1.20% in order to reach a maximum score of 100%. The lists showed equivalent results and similar phonetic content in previous studies.^29^, ^30^

The sentences were projected in an open field through a loudspeaker positioned in front of the subjects (0° azimuth), one meter away, at a sound pressure level (dB SPL) of 65 decibels. The result was expressed as speech recognition index (SRI), which corresponds to the number of words repeated correctly in each sentence, multiplied by the correction value, and expressed in percentage. The SRI score ranged from 0 (a poor performance) to 100% (the best performance).

### Statistical analysis

eABR wave V amplitudes, latencies and interpeak III–V intervals recorded in the apical, mid, and basal electrodes were compared using the One-way analysis of variance (ANOVA). Pearson’s product-moment correlation coefficient was adopted to investigate associations between intraoperative eABRs and subjects’ behaviorally determined thresholds and SRI scores obtained from them after 6 months of CI use. The results were expressed in coefficient correlation (r) and confidence intervals (Confidence Interval 95% = lower limit; upper limit). A significance level of 5% was adopted.

## Results

Positive eABRs were recorded in 20 of 21 subjects during CI surgery. For one subject (#21), the recording was classified as negative eABRs since wave V could not be identified in two replications of the same stimuli for the three tested electrodes. For all subjects, waves I and II could not be recorded due to the presence of stimulus artifact.

**Figure 3:**
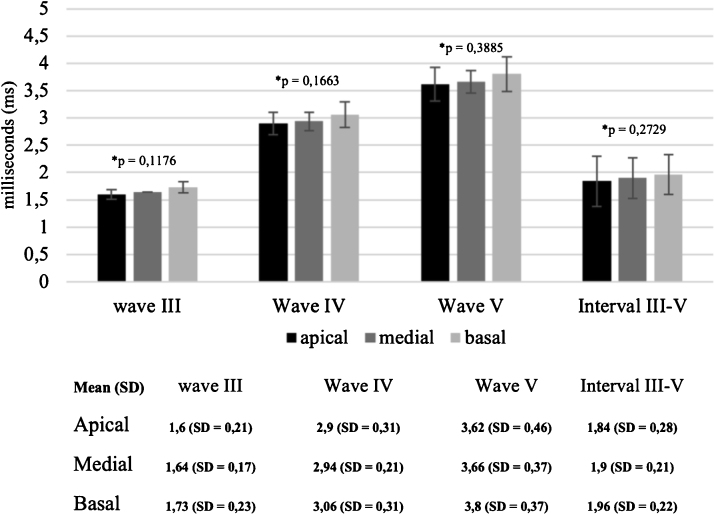
Mean waves latencies and interpeak III–V interval registered in the apical, mid, and basal electrodes, during CI surgery. The bars indicate the standard deviation. The one-way analysis of variance (ANOVA) showed that there are no significant differences between waves latencies across the three different regions of the cochlea (*p* > 0.05).

Fig. 3 shows the mean waves latencies and interpeak III–V intervals recorded in apical, mid and basal electrodes. Although absolute waves latencies and interpeak III–V intervals were longer in the basal electrode (e9) compared to the mid (e14) and apical (e19) electrodes, the one-way analysis of variance (ANOVA) showed that these differences were not significant (*p* = 0.3885, *p* = 0.1663, *p* = 0.1176 and *p* = 0.2729, for waves V, IV, III and Interpeak III–V interval, respectively).

The mean wave V amplitudes were, respectively, 0.346 µV (SD = ±0.21 µV), 0.322 µV (SD = ±0.2 µV) and 0.251 µV (SD = ±0.24 µV), for the apical, mid, and basal electrodes. Wave V amplitudes were larger in the apical electrode compared to the mid and basal electrodes, but the one-way analysis of variance (ANOVA) showed that this difference was not significant (*p* =  0.4156).

**Figure 4:**
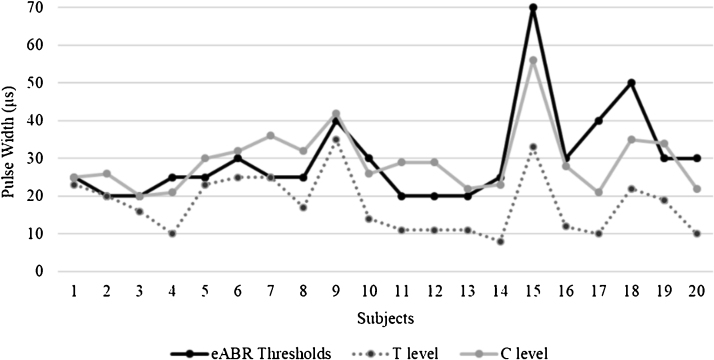
Comparison between eABR thresholds and behavioral levels across subjects.

The eABR thresholds obtained from subjects during CI surgery ranged from 20 to 70 µs (mean = 30.00 ± 12.25 µs). The mean behaviorally determined T- and C-levels of subjects during sound processor activation were 17.75 (SD = ±7.87 µs) and 29.45 (SD = ±8.59 µs), respectively. The Pearson’s product-moment correlation coefficient revealed that they were correlated (Table 2). The mean eABR threshold was near the mean behavioral comfort level of the subjects and it showed a strongest correlation with C-levels compared to the T-levels, however, there was considerable variability in the individual eABR thresholds of the subjects. For eight subjects, eABR thresholds were registered inside the electrical dynamic range, between T- and C-levels (they were more likely at 70% of the dynamic range, close to C-levels), for 2 subjects eABR thresholds and C-levels showed equal values, eight subjects showed eABR thresholds at higher values than C-levels (average of 8.5 µs of pulse duration above the C-levels) and 2 subjects showed eABR thresholds and T-levels at equal values. Despite the variability, eABR thresholds were recorded at audible electrical levels in all subjects included in this study. The Fig. 4 shows the comparison between eABR thresholds and behavioral levels across subjects. The subject #21 was excluded of the analysis since he showed negative eABRs.

**Table 2:** Comparison between intraoperative eABR thresholds and behaviorally determined levels of subjects in the sound processor activation.

Behavioral level	r	95% CI
T-Level	0.54	0.13; 0.79^a^
C-Level	0.74	0.44; 0.89^a^

Behavioral level, Behavioral determined levels from subjects in the sound processor activation; r, coefficient correlation; 95% CI, Confidence Intervals 95% = lower; upper limit.

a Significant correlation, Pearson’s product-moment correlation coefficient, at significance level of 5%.

**Figure 5:**
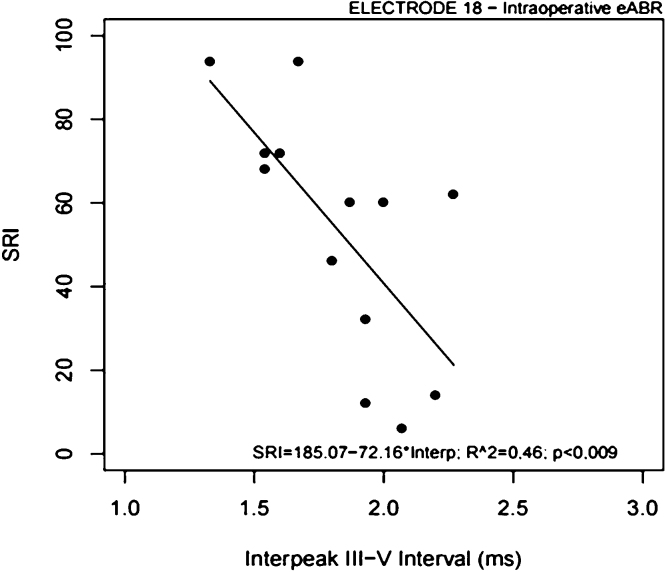
Scatter plot showing the relationship between SRI scores and intraoperative interpeak III–V intervals recorded in the electrode 18. The correlation was significant (*p* < 0.009).

All 21 subjects completed the open-set sentences recognition test after 6-months of CI use. Their SRI scores ranged from 2% to 94% (Median = 60%; Mean = 51.52 ± 31.06%). Table 3 shows the individual SRI scores across subjects. The subject #21 showed negative eABRs in the three tested electrodes (absent eABRs) and the lowest SRI score compared to the group (SRI = 2%) (Table 3). The Pearson’s product-moment correlation coefficient revealed a significant and negative correlation between SRI scores and intraoperative interpeak III–V intervals recorded in the electrode 18 (Table 4; Fig. 5). There were no correlations between SRI scores and interpeak III–V intervals recorded in the electrodes 9 and 14, and between SRI scores and wave V latencies and amplitudes recorded in the electrodes 18, 14 and 9 (Table 4).

**Table 3:** Individual speech recognition index (SRI) across subjects.

Subject	SRI (%)
1	72
2	36
3	6
4	68
5	94
6	90
7	60
8	68
9	62
10	16
11	46
12	90
13	72
14	32
15	12
16	94
17	14
18	60
19	72
20	16
21	2

SRI, number of words repeated correctly in each sentence multiplied by correction factor, expressed in percentage (integer numbers).

**Table 4:** Comparison between wave V amplitudes, latencies and interpeak III–V intervals recorded during cochlear implant surgery and sentences recognition scores obtained from the subjects after six months of cochlear implant use.

	Electrode	r	95% CI
Interpeak III–V Interval (µs)	18	−0.69	−0.9; −0.22^a^
	14	−0.58	−0.86; −0.049
	9	−0.078	−0.58; 0.47
Wave V Latency (µs)	18	−0.46	−0.75; −0.016
	14	−0.41	−0.72; 0.035
	9	−0.22	−0.6; 0.25
Wave V Amplitude (nV)	18	0.57	0.19; 0.081
	14	0.47	0.035; 0.76
	9	0.41	−0.035; 0.72

Electrode: intracochlear electrode selected for e-ABR testing; r, coefficient correlation, 95% CI, Confidence Intervals, 95% = lower.

a Significant correlation, Pearson’s product-moment correlation coefficient, at significance level of 5%.

## Discussion

The first purpose of this study was to investigate intraoperative eABRs recorded in three different electrodes corresponding to input bands with frequencies centered at 500 Hz, 1000 Hz, and 2000 Hz, allocated, respectively, to the electrodes e18, e14, and e9, in the Evo® electrode array. The eABR waveforms showed similar morphology across the different electrode locations. Wave V was the most robust and frequently recorded, followed by waves III, and IV, respectively. Waves I and II could not be recorded due to the presence of stimulus artifact. These findings are in agreement with some earlier studies in adult CI users,^18^, ^26^, ^31^ including the previous generation of Digisonic users, DX10.^16^, ^17^, ^32^, ^33^

The eABR waves latencies and amplitudes were also similar across the different electrode locations. Although latencies and interpeak III–V intervals were shorter and Wave V amplitudes were larger in the apical electrode compared to the mid and basal electrodes, the differences were not significant, and they showed similar standard deviation between them. Similar results were reported by Firszt et al.^9^ for postoperative eABR amplitudes in 11 adult Clarion cochlear implant users. The authors found Wave V latencies significantly longer for the basal electrode compared with the mid and apical electrodes, however, it is controversial in the literature and another study has reported no significant differences on waves latencies across the different electrode locations in users of other CI systems.^26^ There are no published studies comparing the wave V latency across the different electrode locations for Evo® recipients. It is supposed that wave V amplitude is related to the synchrony of auditory nerve fibers responses for electrical stimulation^9^ and wave V latency is related to the activity transmission between neural synapses.^34^ The results of our study suggest that these features may be immediately activated after the placement of the Evo® electrode array inside the cochlea and there are no differences between them regardless the electrode position.

Intraoperative eABR thresholds were significantly correlated with both, behaviorally determined T- and C-levels obtained from subjects during their sound processor activation, and they were recorded at audible electrical stimulation levels for all Evo® recipients included in this study. The mean intraoperative eABR threshold was recorded near the mean subject’s behaviorally determined C- level, at higher value than the previously reported by Guenser et al.^3^ for postoperative eABR in Evo® recipients, despite the considerable variability in the individual eABR thresholds of the subjects.

These results are in accordance with previous studies comparing intraoperative eABR thresholds and behavioral determined thresholds in other CI systems. Shallop et al.^21^ studied the relationship between eABR thresholds obtained from 11 Nucleus device recipients in the operating room and subsequent behavioral measures of threshold and maximum comfort levels. Among 12 adults and 14 children with the Nucleus CI, eABR thresholds were strongly correlated with both T- and C-levels, and they were registered at audible stimulation levels for all subjects. Among subjects for whom both intraoperative and postoperative eABR measures were obtained, intraoperative eABR thresholds were consistently higher than postoperative thresholds.^22^ After CI surgery, variation in the charge delivered to intracochlear electrodes occurs that is attributable to factors such as healing and reduction of the interface between electrode and neural tissue, electrochemical gradient changes within the cochlea, and re-organization and adaptation of the auditory nerve that becomes more conductive to electrical stimulation over time.^35^, ^36^ Therefore, intraoperative eABR thresholds would be recorded at higher values compared with postoperative thresholds because of the need for a higher CI stimulation energy to elicit a recordable action potential from brainstem at this time.

The results of this study showed that intraoperative eABR thresholds may not be used to predict either T- or C-levels, but they can be used to establish an audible level for Evo® recipients. There are no previous studies comparing the intraoperative eABR thresholds and behaviorally determined thresholds in Evo® recipients and this information is relevant for programming the sound processor during its activation. In the sound processor activation, the stimulation levels are currently determined through behavioral measures, based on the response of CI users. For young children or patients with special needs, as well as for newly implanted patients and those with no previous experience with electrical stimulation promoted by cochlear implant, determining the optimal psychophysical levels of electrical stimulation is still a challenge. Additionally, the use of the intraoperative eABR recordings has the advantage to be obtained in subjects while under general anesthesia, minimizing the interference of muscular artifacts that could hinder the measurement.

There was a significant correlation between intraoperative eABR and speech perception of the Evo® users after 6-months of device use. Interpeak III–V intervals recorded in the apical electrode (electrode 18) were negatively correlated to the sentence recognition scores of the subjects. It suggests that longer interpeak III–V intervals in the apical region of the cochlea (500 Hz), may be related to inferior auditory performance with CI use in short-term. Our results are consistent with those of Gallégo et al.^16^, ^17^ for postoperative eABRs in Digisonic DX10 users. According to the authors, these findings could be explained by the influence of decoding processing from the cochlear nucleus level and the degeneration of the auditory system. Degeneration of the auditory system indicates demyelination which could increase the propagation time of nerve impulses between the cochlear nucleus and the inferior colliculus, thereby decreasing speech intelligibility performance. According to the authors, the longer the interpeak III–V interval, the worse the speech intelligibility.^16^ Lundin et al.^15^ also found significant correlations between speech perception outcomes in CI recipients and eABR characteristics solely for electrodes in the low-frequency region of the cochlea. Gordon et al.^37^ reported that humans with hearing loss have larger populations of spiral ganglion cells in the cochlear apex (low-frequency region) than in the cochlear base (high-frequency region) and that speech perception may depend on auditory pathway encoding, which is based on nerve survival and synchronous neural activity; this may explain our results in the apical electrode.

One subject showed absent eABRs (negative eABRs), and he also showed the poorest speech performance compared to the group after 6-months of CI use. The subject #21 had hearing loss caused by meningitis and it could explain the poor speech scores obtained from this subject in our study. It is well known that meningitis impacts the cochlea and the central nervous system despite complete electrode insertion.^38^, ^39^, ^40^, ^41^

Similar findings were previously reported in the literature. Lundin et al.^15^ carried out a retrospective study of 74 adults and four children with severe cochlear abnormalities who received CIs between 2011 and 2013 reported that absent intraoperative eABRs always predicted low postoperative speech perception scores. Firszt et al.^9^ studied 11 adult subjects implanted with Clarion CI and showed that subjects without open-set speech recognition demonstrated poor morphology or absent eABRs, and subjects with higher values of open-set recognition demonstrated the presence of eABRs. Yamazaki et al.^42^ studied 19 children with cochlear nerve deficiency who underwent a cochlear implantation and intraoperative eABR testing and showed that the presence or absence of eABR recordings was significantly associated with postoperative auditory performance. These authors showed that the mean category of auditory performance in subjects in whom eABRs were present was significantly higher than the main category of auditory performance in subjects with absent eABRs.

Thus, the results of this study can help professionals to plan further actions aiming to improve the auditory performance of CI recipients such as a regular followup, more intensive speech-language therapy, or counseling caregivers, once the longer interpeak III–V interval or an absent intraoperative eABR could suggest inferior auditory performance of CI users in the short term.

Finally, this study was carried out with a small sample size (n = 21), nevertheless, it was possible to detect statistically significant associations between eABR interpeak III–V intervals and sentence recognition scores of the subjects in the short term, using the Pearson’s product-moment correlation coefficient at significance level of 5%. Further investigations with a larger number of subjects are required to explore these results also in the long term. Additionally, although the mean eABR threshold was recorded near the mean C-level of the patients, we found considerable variability in the individual eABR thresholds of the subjects, and, in some cases, the eABR thresholds exceeded the behavioral C-levels of them (average of 8.5 µs of pulse duration). These results can help professionals to stablish audible levels for the first fitting of the speech processor in Evo® recipients, associated with behavioral measures that ensure that these levels are not uncomfortable for them.

## Conclusion

Intraoperative eABR measure proved to be an effective tool for clinical use in Evo® CI electrode array recipients. eABR thresholds can be used to establish audible levels for fitting the sound processor in Evo® recipients and the longer interpeak III–V interval or absent intraoperative eABRs could be related to inferior auditory performance of CI users in the short term. It could help professionals in planning further actions aimed to improve the auditory performance of CI recipients.

## Conflicts of interest

The authors declare no conflicts of interest.

## References

[1] Firszt J.B., Holden L.K., Skinner M.W., Tobey E.A., Peterson A., Gaggl W. (2004). Recognition of speech presented at soft to loud levels by adult cochlear implant recipients of three cochlear implant systems. Ear Hear.

[2] Moberly A.C., Bates C., Haris M.S., Pisoni D.B. (2016). The enigma of poor performance by adults with cochlear implants. Otol Neurotol.

[3] Guenser G., Laudanski J., Phillipon B., Backus C.B., Bordure P., Romanet P. (2014). The relationship between electrical auditory brainstem responses and perceptual thresholds in Digisonic® SP cochlear implant users. Cochlear Implants Int.

[4] Gordon K.A., Papsin B.C., Harrison R.V. (2004). Toward a battery of behavioral and objective measures to achieve optimal cochlear implant stimulation levels in children. Ear Hear.

[5] Basta D., Dahme A., Todt I., Ernst A. (2007). Relationship between intraoperative eCAP thresholds and postoperative psychoacoustic level as a prognostic tool in evaluating the rehabilitation of cochlear implantees. Audiol Neurotol.

[6] Greisiger R., Shallop J.K., Hol P.K., Elle O.J., Jablonski G.E. (2015). Cochlear implantees: analysis of behavioral and objective measures for a clinical population of various age groups. Cochlear Implants Int.

[7] de Vos J.J., Biesheuvel J.D., Briaire J.J., Boot P.S., van Gendt M.J., Dekkers O.M. (2018). Use of electrically evoked compound action potentials for cochlear implant fitting: a systematic review. Ear Hear.

[8] Liebscher T., Alberter K., Hoppe U. (2018). Cortical auditory evoked potentials in cochlear implant listeners via single electrode stimulation in relation to speech perception. Int J Audiol.

[9] Firszt J.B., Chambers R.D., Kraus N. (2002). Neurophysiology of cochlear implant users II: comparison among speech perception, dynamic range, and physiological measures. Ear Hear.

[10] Tysome J.R., Axon P.R., Donnelly N.P., Evans G.D., Ferner R.E., Fitzgerald O’Connor A.F. (2013). English consensus protocol evaluating candidacy for auditory brainstem and cochlear implantation in neurofibromatosis type 2. Otol Neurotol.

[11] Brown C.J., Abbas P.J., Bertschy M., Tyler R.S., Lowder M., Takahashi G. (1995). Longitudinal assessment of physiological and psychophysical measures in cochlear implant users. Ear Hear.

[12] Groenen P.A.P., Makhdoum M., Van Den Brink J.L., Stollman M.H.P., Snik A.F.M., Van Den Broek P. (1996). The relation between electric auditory brain stem and cognitive responses and speech perception in cochlear implant users. Acta Otolaryngol.

[13] Walton J., Gibson W.P., Sanli H., Prelog K. (2008). Predicting cochlear implant outcomes in children with auditory neuropathy. Otol Neurotol.

[14] Gibson W.P.R., Sanli H., Psarros C. (2009). The use of intra-operative electrical auditory brainstem responses to predict the speech perception outcome after cochlear implantation. Cochlear Implants Int..

[15] Lundin K., Stillesjo F., Hask-Andersen H. (2015). Prognostic value of electrically evoked auditory brainstem responses in cochlear implantation. Cochlear Implants Int.

[16] Gallego S., Truy K., Morgon A., Collet L. (1997). EABRS and surface potentials with a transcutaneous multielectrode cochlear implant. Acta Otolaryngol.

[17] Gallego S., Frachet B., Micheyl C., Truy E., Collet L. (1998). Cochlear implant performance and electrically-evoked auditory brain-stem response characteristics. Electroencephalogr Clin Neurophysiol.

[18] Shallop J.K., Beiter A.L., Goin D.W., Mischke R.E. (1990). Electrically evoked auditory brain stem responses (EABR) and middle latency responses (EMLR) obtained from patients with the nucleus multichannel cochlear implant. Ear Hear.

[19] Brown C.J., Hughes M.L., Luk B., Abbas P.J., Wolaver A., Gervais J. (2000). The relationship between EAP and EABR thresholds and levels used to program the nucleus 24 speech processor: data from adults. Ear Hear.

[20] Gordon K.A., Papsin B.C., Harrison R.V. (2003). Activity-dependent developmental plasticity of the auditory brain stem in children who use cochlear implants. Ear Hear.

[21] Shallop J.K., VanDyke L., Goin D.W., Mischke R.E. (1991). Prediction of behavioral threshold and comfort values for nucleus 22-channel implant patients from electrical auditory brainstem response test results. Ann Otol Rhinol Laryngol.

[22] Brown C.J., Abbas P.J., Fryauf-Bertschy H., Kelsay D., Gantz B.J. (1994). Intraoperative and postoperative electrically evoked auditory brain stem responses in nucleus cochlear implant users: implications for the fitting process. Ear Hear.

[23] Undurraga J.A., Carlyon R.P., Macherey O., Wouters J, van Wieringen A. (2012). Spread of excitation varies for different electrical pulse shapes and stimulation modes in cochlear implants. Hear Res.

[24] Martins G.D.S.Q., Neto R.V.B., Tsuji R.K., Gebrim E.M.M.S., Bento R.F. (2015). Evaluation of intracochlear trauma caused by insertion of cochlear implant electrode arrays through different quadrants of the round window. BioMed Res Int.

[25] Picton T.W., Hillyard S.A. (1974). Human auditory evoked potentials. II. Effects of attention. Electroencephalogr Clin Neurophysiol.

[26] Abbas P.J., Brown C.J. (1988). Electrically evoked brainstem potentials in cochlear implant patients with multi-electrode stimulation. Hear Res.

[27] Marozeau J., Ardoint M., Gnansia D., Lazard D. (2018). Acoustic match to electric pulse trains in single-sided deafness cochlear implant recipients. Proc Int Symp Audit Audiol Res.

[28] Costa M.J., Santos S.N., Lessa A.H., Mezzomo C.L. (2015). Proposal for implementing the Sentence Recognition Index in individuals with hearing disorders. Codas.

[29] Costa M.J., Iório M.C.M., Albernaz P.L.M. (2000). Development of a test to evaluate speech recognition with and without noise. Pró-fono.

[30] Santos S.N., Daniel R.C., Costa M.J. (2009). Study of equivalence among the lists of sentences in Portuguese. Revista CEFAC.

[31] Abbas P.J., Brown C.J. (1991). Electrically evoked auditory brainstem response: growth of response with current level. Hear Res.

[32] Gallego S., Garnier S., Micheyl C., Truy E., Morgon A., Collet L. (1999). Loudness growth functions and EABR characteristics in Digisonic cochlear implantees. Acta Otolaryngol.

[33] Truy E., Gallego S., Chanal J.M., Collet L., Morgon A. (1998). Correlation between electrical auditory brainstem response and perceptual thresholds in Digisonic cochlear implant users. Laryngoscope.

[34] Eggermont J.J. (1988). On the rate of maturation of sensory evoked potentials. Electroencephalogr Clin Neurophysiol.

[35] Shallop J.K., Ash K.R. (1995). Relationships among comfort levels determined by cochlear implant patient’s self-programming, audiologist’s programming, and electrical stapedius reflex thresholds. Ann Otol Rhinol Laryngol.

[36] Brown C.J. (2003). Clinical uses of electrically evoked auditory nerve and brainstem responses. Curr Opin Otolaryngol Head Neck Surg.

[37] Gordon K.A., Papsin B.C., Harrison R.V. (2007). Auditory brainstem activity and development evoked by apical versus basal cochlear implant electrode stimulation in children. Clin Neurophysiol.

[38] Hugosson S., Carlsson E., Borg E., Brorson L.O., Langeroth G., Olcén P. (1997). Audiovestibular and neuropsychological outcome of adults who had recovered from childhood bacterial meningitis. Int J Pediatr Otorhinolaryngol.

[39] Wellman M.B., Sommer D.D., McKenna J. (2003). Sensorineural hearing loss in postmeningitic children. Otol Neurotol.

[40] Schmidt H., Heimann B., Djukic M., Mazurek C., Fels C., Wallesch C.-W. (2006). Neuropsychological sequelae of bacterial and viral meningitis. Brain.

[41] Grasel S., Greters M., Goffi-Gomez M.V.S., Bittar R., Weber R., Oiticica J. (2018). P3 cognitive potential in cochlear implant users. Int Arch Otorhinolaryngol.

[42] Yamazaki H., Leigh J., Briggs R., Naito Y. (2015). Usefulness of MRI and EABR testing for predicting CI outcomes immediately after cochlear implantation in cases with cochlear nerve deficiency. Otol Neurotol.

